# Postmortem diagnosis of very long chain acyl‐CoA dehydrogenase (VLCAD) deficiency in a neonate with sudden cardiac death

**DOI:** 10.1002/jmd2.12365

**Published:** 2023-04-30

**Authors:** Prapti Singh, Deirdre Amaro, Olugbemisola Obi, FNU Kiran, Erin Hediger, Tomi L. Toler, Patricia I. Dickson, Dorothy K. Grange

**Affiliations:** ^1^ Division of Genetics and Genomic Medicine, Department of Pediatrics Washington University School of Medicine in St. Louis Saint Louis Missouri USA; ^2^ Division of Reproductive Endocrinology and Infertility, Department of Obstetrics and Gynecology University of Iowa, Roy J. and Lucille A. Carver College of Medicine Iowa City Iowa USA; ^3^ Department of Pathology and Anatomical Sciences University of Missouri School of Medicine Columbia Missouri USA; ^4^ Division of Neonatology, Department of Child Health University of Missouri School of Medicine Columbia Missouri USA

**Keywords:** cardiac arrest, newborn screening, very long chain acyl CoA dehydrogenase (VLCAD) deficiency

## Abstract

Very long chain acyl‐CoA dehydrogenase (VLCAD) deficiency is an autosomal recessive long chain fatty acid β‐oxidation disorder with a variable clinical spectrum, ranging from an acute neonatal presentation with cardiac and hepatic failure to childhood or adult onset of symptoms with hepatomegaly or rhabdomyolysis provoked by illness or exertion. Neonatal cardiac arrest or sudden unexpected death can be the presenting phenotype in some patients, emphasizing the importance of early clinical suspicion and intervention. We report a patient who had a cardiac arrest and died at one day of age. Following her death, the newborn screen reported biochemical evidence of VLCAD deficiency, which was confirmed with pathologic findings at autopsy and by molecular genetic testing.

**Synopsis**

The risk of neonatal cardiac complications and sudden death associated with VLCAD deficiency in this case demonstrates the importance of early detection and diagnosis. Cardiac evaluation, monitoring and institution of treatment may alter the prognosis.

## 1 INTRODUCTION

Newborn screening is advantageous for patients with inborn errors of metabolism by providing early intervention with nutritional and medical treatment to improve the long‐term outcomes. However, these interventions can be of limited benefit in cases with a severe and early onset presentation.

Very long chain acyl‐CoA dehydrogenase (VLCAD) deficiency, caused by variants in *ACADVL*, (MIM 201475) is a long chain fatty acid β‐oxidation disorder, which results in an inability to metabolize long‐chain fatty acids with a chain length of 14–20 carbons. The phenotypic spectrum is broad and can range from an acute neonatal presentation with multi‐organ system involvement to a childhood or adult onset presentation with hepatomegaly or rhabdomyolysis provoked by states of stress.^1^

Following the institution of expanded newborn screening in the United States, the number of infants identified with *ACADVL* variants has increased, and data regarding genotype/phenotype correlation has expanded.^2^, ^3^, ^4^

Intervention for VLCAD deficiency typically involves high dextrose concentration fluids during acute management, and maintenance therapy with medium chain triglyceride (MCT) supplementation, provision of formula with high MCT and low long chain triglyceride content, and other dietary modifications.^5^, ^6^, ^7^, ^8^, ^9^ Triheptanoin was recently approved for use in VLCAD deficiency and may be given in place of MCT.^10^ Gene therapy is being explored as an option for treatment.^11^, ^12^ Intervention in infants with fatty acid oxidation defects who present with an acute cardiac event is often unsuccessful, with a high incidence of death.^13^ We report a neonate with VLCAD deficiency diagnosed postmortem following a cardiac arrest at one day of age.

## 2 CASE REPORT

The patient was a female infant born at 40 weeks gestation to a 26‐year‐old gravida 1, para 0 mother by vaginal delivery following an uncomplicated pregnancy. Birth weight was 3340 g. APGAR scores were 9 and 9 at 1 and 5 min, respectively, and she was transitioned to routine neonatal care. A few grunting episodes were noted on the first day of life but resolved spontaneously. She was being breastfed every 3–4 h since birth without difficulty. At 30 h of age, 2 h after the last feeding, she became unresponsive, resulting in resuscitation efforts by neonatology and pediatric cardiology. An echocardiogram showed no structural abnormalities. Continuous cardiac monitoring showed a pattern of wide QRS complexes with peaked T waves refractory to intervention. Initial venous blood obtained after 1 h of resuscitation showed pH 7.002 and with a base deficit of 10. Initial potassium was >10 mmol/L. Resuscitation efforts were discontinued after 2 h. Newborn screening blood spot samples had been collected ~6 h prior to the cardiac arrest. The newborn screen report was received 2 days after the infant died and indicated a positive screen for VLCAD deficiency with C14 of 5.07 μmol/L (normal <0.70 μmol/L), C14:1 of 3.20 μmol/L (normal <0.60 μmol/L), C14:2 of 0.30 μmol/L (normal <0.10 μmol/L), C14:1/C12:1 of 9.13 μmol/L (normal <3.0 μmol/L), and C14:1/C16 of 0.25 μmol/L (normal <0.20 μmol/L).

**FIGURE 1:**
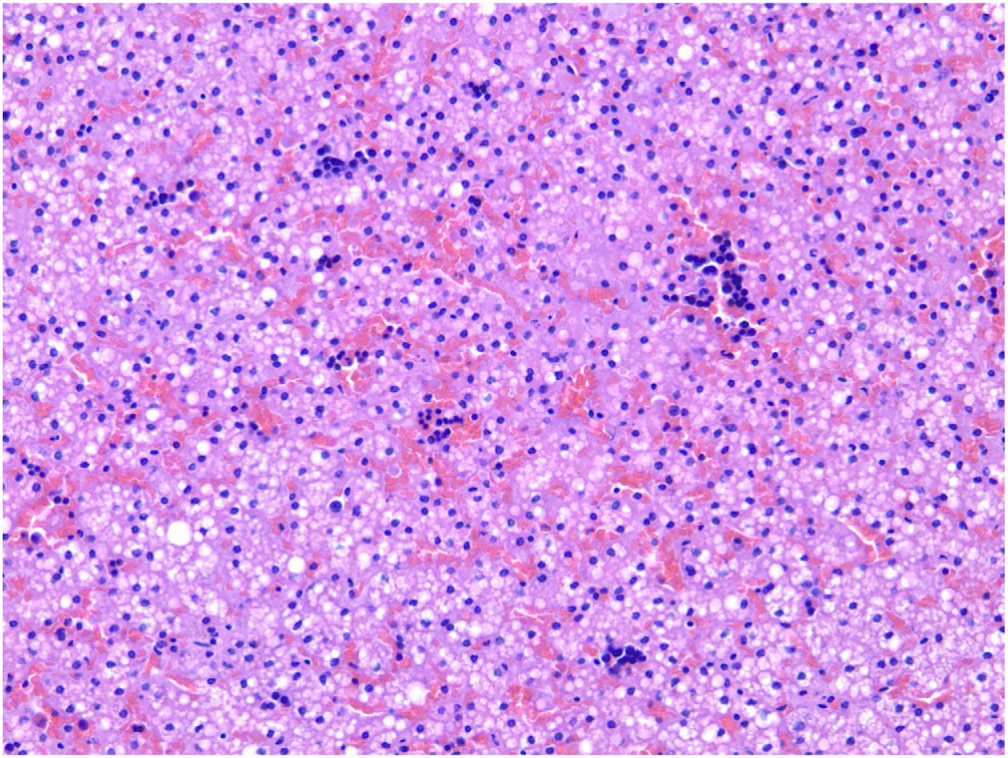
Postmortem microscopic examination of the liver demonstrating microsteatosis (H&E staining).

Postmortem examination was performed and the parents consented to genetic testing. Examination of the placenta was notable for rare pigmented macrophages concerning for meconium present in utero. Autopsy findings revealed a structurally normal heart without hypertrophy, evidence of meconium aspiration on examination of the lungs, early ischemic changes in the stomach, renal tubular vacuolization, and microsteatosis of the liver (Figure 1).

Chromosome microarray analysis from a postmortem sample was normal. Given the patient's acute and unexpected death, exome sequencing was performed and revealed biallelic variants in *ACADVL* [maternally inherited c.1375dup, p.R459Pfs*4 pathogenic variant and paternally inherited c.1678 + 3_1678 + 6del, p.? likely pathogenic variant]. The maternal variant is predicted to result in nonsense‐mediated decay, and the paternal variant is predicted to have a deleterious effect on splicing. These variants are consistent with the loss of function mechanism of disease in VLCAD deficiency. No other variants were identified on the sequencing study.

## 3 DISCUSSION

This case of severe neonatal VLCAD deficiency presented as cardiac arrhythmia and subsequent cardiac arrest. Approximately 81% of pathogenic truncating variants result in a severe early onset form of this condition, while milder childhood and adult forms are typically associated with some residual enzyme activity.^14^ A previous report describes the cardiac phenotype associated with various genotypes.^15^ More recent literature has shown that genotype–phenotype correlation is less predictable with the expanding number of variants being reported.^2^, ^3^, ^4^, ^13^ The novel combination of heterozygous *ACADVL* variants in this patient, coupled with her acute presentation, contributes important data regarding clinical phenotypes associated with this condition.

Although milder forms of VLCAD deficiency are more common, the possibility of early onset cardiac or multiorgan system failure warrants prompt intervention and monitoring for all cases detected through newborn screening. This patient's early acute presentation serves as an argument to collect newborn screening samples sooner rather than as late as 72 h after birth, as is the policy for some newborn screening programs. In the state of Missouri, the newborn screening sample is collected between 24 and 48 h of age. Even with early sample collection, the results may not be reported soon enough to identify severely affected infants before they have become symptomatic or may already have died suddenly.

Cardiac arrhythmia can be the presenting symptom in VLCAD deficiency and tragically, may be fatal, as reported in one study showing mortality between the ages of 3.5 and 9 months among 5 patients.^13^ The neonatal acute cardiac presentation resulting in demise has been well described in the literature; these studies included metabolic and molecular genetic test results, as well as pathologic findings.^16^, ^17^, ^18^ The hepatic steatosis found in the current case is believed to be secondary to VLCAD deficiency, but the gastric and renal pathologic findings were likely secondary to the prolonged resuscitation efforts. Meconium aspiration was a coexistent finding that could have contributed to the patient's symptoms, but was unlikely to have precipitated her acute decompensation.

Due to an acute clinical presentation prior to availability of screening results, or lack of newborn screening in some reported cases, it may not be possible to implement intervention.^17^, ^18^, ^19^ However, intervention with dextrose containing intravenous fluids and MCTs may alter the prognosis if initiated early enough postnatally.^16^ Scalais et al. reported a family in which the first two children died of sudden unexplained cardiopulmonary arrest at less than 36 hours of age.^16^ Therefore, with a subsequent pregnancy, the infant was electively admitted immediately after birth to the neonatal intensive care unit and underwent diagnostic testing that revealed VLCAD deficiency; preemptive treatment prevented cardiac complications and a fatal outcome, and the infant survived.^16^ Acylcarnitine analysis on newborn screening blood spot samples retrieved from the deceased siblings was consistent with VLCAD deficiency.

In conclusion, we report a case of VLCAD deficiency presenting with cardiac arrest soon after birth, with postmortem confirmatory genetic testing and hepatic steatosis identified at autopsy. Increased awareness of a more severe presentation of VLCAD deficiency could potentially alter the prognosis for some cases. Additionally, postmortem testing is vital to make an accurate diagnosis and to assist with genetic counseling, family planning and early neonatal intervention in future pregnancies.

## AUTHOR CONTRIBUTIONS

Prapti Singh participated in literature review, patient data acquisition, data interpretation and manuscript preparation. Deirdre Amaro and Olugbemisol Obi participated in patient data acquisition, data interpretation, consent and manuscript preparation. FNU Kiran participated in patient data acquisition and data interpretation. Erin Hediger participated in patient data acquisition and interpretation. Tomi L. Toler participated in patient data acquisition and manuscript preparation. Patricia Dickson and Dorothy K. Grange participated in manuscript preparation.

## FUNDING INFORMATION

None.

## CONFLICT OF INTEREST STATEMENT

Patricia Dickson declares research support from Genzyme, Alnylam, and M6P Therapeutics. She is also a consultant for Mandos Health. All other authors declare no conflicts of interest.

## ETHICS STATEMENT

This article does not contain any studies with human or animal subjects performed by any of the authors. All procedures followed were in accordance with the ethical standards of the responsible committee on human experimentation (institutional and national) and with the Helsinki Declaration of 1975, as revised in 2000 (5). This single case study is IRB exempt at our institution.

## INFORMED CONSENT

Informed consent was obtained from the parents for inclusion of the patient in the study. Proof that informed consent was obtained is available upon request. If doubt exists whether the research was conducted in accordance with the Helsinki Declaration, the authors will explain the rationale for their approach, and demonstrate that the institutional review body explicitly approved the doubtful aspects of the study.

## Data Availability

Data and material were accessible through institutional medical records at the respective institutions.
